# Correlation between Intraoperative Fluid Administration and Outcomes of Pancreatoduodenectomy

**DOI:** 10.1155/2020/8914367

**Published:** 2020-07-30

**Authors:** Xuefeng Cao, Xixiu Wang, Baolei Zhao, Lingqun Kong, Lei Zhou, Wentao Zhu, Xutao Lin, Qiangpu Chen, Xingyuan Zhang

**Affiliations:** ^1^Department of Hepatobiliary Surgery, Binzhou Medical University Hospital, Binzhou, Shandong Province, China; ^2^Department of Cardiovascular Medicine, Binzhou Medical University Hospital, Binzhou, Shandong Province, China

## Abstract

**Background:**

Intraoperative fluid (IOF) administration plays an important role during major abdominal surgery although increased fluid intake can adversely influence postoperative outcomes. However, the effect of the IOF rate on the outcomes of pancreatoduodenectomy (PD) is unclear.

**Methods:**

151 patients, who underwent PD at Binzhou Medical University Hospital between January 2010 and May 2017, were categorized into three groups according to IOF rates (ml/kg/hr): restricted (<10, *n* = 47), standard (10–15, *n* = 76), and liberal (>15, *n* = 28).

**Results:**

The overall postoperative morbidity was 56.95%. The incidence of postoperative pancreatic fistula (POPF) was 11.26%. The in-hospital mortality rate was 7.28% with the most common cause being grade C POPF and secondary intra-abdominal infections. The patients in the liberal group had significantly higher incidences of POPF (25%) and respiratory complications (21.43%). The other outcome parameters such as recovery of bowel function, hospital stay, and postoperative daily drainage were similar among the groups. Multivariable analysis confirmed the IOF rate to be most strongly associated with POPF (odds ratio: 5.195, confidence interval: 1.142–23.823, *P* = 0.023) and respiratory complications (odds ratio: 7.302, confidence interval: 0.676–58.231, *P* = 0.025).

**Conclusions:**

The IOF rate significantly affects the incidence of POPF and respiratory complications after PD. Careful patient-oriented fluid therapy may help to prevent these complications.

## 1. Introduction

Fluid administration, especially of intraoperative fluid (IOF), is an integral part of almost any surgical procedure [1]. During operations, there are third space losses with peripheral vasodilatation due to anesthesia. Fluid loading with large amounts of intravenous (IV) fluids is done in clinical practice to expand the intravascular space and improve organ perfusion [2]. However, several studies [3–8] suggest that perioperative fluid administration (especially IOF administration) has a substantial direct impact on the outcomes in patients undergoing major abdominal surgery. Several randomized controlled trials (RCTs) and meta-analyses concerning perioperative fluid management strategies in major operations such as colorectal surgery have found that perioperative fluid restriction is associated with enhanced recovery of gastrointestinal function, and reduced complications and hospital stay [1, 9–11].

Pancreatoduodenectomy (PD) is a complex procedure associated with long operative time, potentially large-volume blood loss, and high surgical morbidity. The incidence of surgical morbidities or complications is 38%–58%—the most common being postoperative pancreatic fistula (POPF), biliary fistula, wound infection, postoperative intraperitoneal hemorrhage, and respiratory complications [12, 13].

During the last 60 years, postoperative mortality has markedly decreased because of improvements in surgical techniques, better perioperative management with early recognition and treatment of complications. However, morbidity after PD remains high [14, 15]. Advanced age, poor nutritional status, high preoperative serum bilirubin, and soft pancreas are important risk factors for mortality and complications after PD [16]. In addition to these factors, we hypothesize that perioperative fluid administration (especially IOF administration) may be another independent factor to influence the outcomes following PD. The results of previous studies on this issue have been controversial [17–22]. Given this uncertainty, we performed this study to determine the correlation between the IOF administration and outcomes following PD.

## 2. Patients and Methods

The study was approved by the Institutional Ethics Committee of Binzhou Medical University Hospital. All the methods in the study were performed in accordance with the relevant regulations in our hospital. Informed consent of patients was not taken separately as this is a retrospective review not revealing patient identities.

### 2.1. Patient Selection

In this study, consecutive patients who underwent PD for benign or malignant pancreatic or periampullary diseases from January 1, 2010, to May 31, 2017, at Binzhou Medical University Hospital were included. Patients with malignant tumors who received neoadjuvant therapy or vascular resection were excluded.

### 2.2. Perioperative Care

All the patients received general anesthesia including drugs for premedication, induction, and maintenance of general anesthesia. As a standard practice, the fluids were used in the ratio of 3 : 1 (crystalloid : colloid). The rate of IOF was at the discretion of the anesthesiologists. Blood transfusions were given when hemoglobin levels were <8 g/dl. Blood was given with crystalloid at a ratio of 1 : 3, and colloid at a ratio of 1 : 1. The details about IOF administration including the fluid type (crystalloid, colloid, or blood product) and the amount of fluid used were recorded.

For this study, we used the IOF rate (ml/kg/hr)—intraoperative infusion volume divided by the duration of the operation and the weight of the patient (instead of the simple volume)—to analyze the fluid management precisely. Similarly, POF rate (ml/kg/hr)—postoperative infusion volume divided by 24 hr and the weight of the patient—was used to analyze postoperative fluid management.

All the patients who underwent PD were categorized into three groups according to the IOF rate: restricted (<10 ml/kg/hr), standard (10–15 ml/kg/hr), and liberal (>15 ml/kg/hr). The cut-off values used for these three groups were based on the current anesthesia guidelines for IOF administration (10–15 ml/kg/hr) [23–25]. The daily drainage of the first three postoperative days was expressed as drainage on POD1, POD2, and POD3. Similarly, POF rate on the first three postoperative days was expressed as the rate of POF1, POF2, and POF3.

### 2.3. Surgical Procedure

All the PD procedures were performed by the same surgical team. An upper midline abdominal incision was made in all cases. The procedure for PD and the digestive tract reconstruction was the same in all patients. A duct to mucosa pancreatojejunostomy was constructed using 5-0 PDS/prolene sutures. According to pancreatic duct size, the corresponding diameter of pancreatic duct stent was placed in the anastomosis. Postoperative care included intravenous antibiotics, maintenance intravenous fluids, and parenteral nutrition support.

### 2.4. Data Collection

Most data were collected from our clinical database including age, gender, and weight of patients; duration of operation; intraoperative infusion volume (crystalloid, colloid, and blood product); estimated intraoperative blood loss; volume of postoperative fluid (POF) administration on the first three days; postoperative outcomes such as the daily drainage on the first three postoperative days; recovery of bowel function; intensive care unit (ICU) admission; postoperative length of hospital stay; and postoperative complications. Intraoperative infusion volume, duration of operation, and estimated intraoperative blood loss data were obtained from the anesthesia records; POF volume and daily drainage of the first three postoperative days were obtained from nursing records; and surgical outcome data were obtained from our clinical records.

### 2.5. Definitions

ISGPS [26] criteria were used for the diagnosis and grading of POPF.

Chylous fistula was diagnosed based on the clinical manifestations and chylous test [27].

Postoperative biliary fistula was diagnosed based on the bilirubin levels in the drain fluid or on the postoperative contrast study.

Postpancreatectomy hemorrhage and delayed postoperative gastric emptying were diagnosed and graded according to the ISGPS criteria [28, 29].

### 2.6. Statistical Analysis

The continuous variables were expressed as the mean and standard deviation (SD). The continuous and categorical variables were compared using ANOVA and Chi-squared tests, respectively. The multivariate analysis was conducted by logistic regression model. All the analyses were performed with Statistical Package for Social Sciences (SPSS) version 24.0 software (IBM Co, Armonk, NY, USA). A *P* value <0.05 was considered to be statistically significant.

## 3. Results

### 3.1. Baseline Characteristics

168 PDs were performed during the study period; 17 patients who underwent PD combined with vascular resection were excluded. Finally, 151 patients (92 males, 59 females) with a mean age of 58.52 years were included in the study and underwent PD. The mean weight of the patients was 62.78 kg (range 45 to 100 kg). There were 47 (31.13%), 76 (50.33%), and 28 (18.54%) patients in the restricted, standard, and liberal groups, respectively. The malignant pathologies included ampullary adenocarcinoma (*n* = 38, 25.17%), cholangiocarcinoma (*n* = 35, 23.18%), duodenal adenocarcinoma (*n* = 30, 19.87%), and pancreatic adenocarcinoma (*n* = 26, 17.22%). The mean duration of operation was 6.12 hours (range 2.90 to 11.33 hours). There were no statistical differences in age, sex ratio, preoperative serum albumin (ALB) level, and body mass index (BMI) among the three groups (*P* > 0.05, Table 1).

### 3.2. Perioperative Fluid Administration

The IOF volume ranged from 2600 to 8100 ml. The mean IOF rate was 11.89 ml/kg/hr (range 4.85 to 21.43 ml/kg/hr). Sixteen patients (10.60%) suffered from excessive bleeding and hypotension and required blood transfusion. The mean volume of blood transfusion was 500 ml. The mean POF rate on the first three postoperative days was 2.08, 2.04, and 2.04 ml/kg/hr, respectively (Table 2).

### 3.3. Postoperative Outcomes

The overall postoperative morbidity was 56.95% with 55 patients suffering from at least one complication. The incidence of POPF was 11.26%, while the incidence of biliary fistula was 5.96%. The in-hospital mortality rate was 7.28% with 54.55% caused by grade C POPF and secondary intra-abdominal infections. The mean postoperative hospital stay was 19.78 ± 9.79 days. The mean duration of recovery of bowel function was 4.66 ± 1.35 days. The daily drain outputs on POD1-POD3 were 133 ± 112 ml, 105 ± 162 ml, and 72 ± 150 ml, respectively. Details of the postoperative outcomes are listed in Table 2.

### 3.4. Comparison of Outcomes among the Three Groups

Intraoperative characteristics and postoperative outcomes among the three groups are compared in Table 2. The pancreatic fistula rate and respiratory complications of the liberal group patients were significantly higher than restricted group patients (*P* = 0.035, 0.025, respectively). There was no statistically significant difference in the other outcomes such as volume of blood transfusion, postoperative length of stay, biliary fistula, postoperative drain output, and postpancreatectomy hemorrhage among the groups (*P* > 0.05).

On multivariable analysis, in addition to plasma ALB levels and BMI of patients, IOF was also a factor significantly associated with POPF and respiratory complications. Additionally, POPF was significantly associated with respiratory complications. The multivariable analysis revealed that the IOF rate was an independent factor associated with POPF (odds ratio: 5.195, confidence interval: 1.142–23.823, *P* = 0.023) and respiratory complications (odds ratio: 7.302, confidence interval: 0.676–58.231, *P* = 0.025) (Table 3).

## 4. Discussion

In recent years, several studies have found that the IOF rate can influence postoperative outcomes after major abdominal surgery. PD is associated with significant morbidity and mortality [30]. It is the touchstone to demonstrate the relationship between IOF administration and the postoperative outcomes. However, the data on IOF administration and post PD outcomes are sparse and controversial.

In the current study, we found that the liberal fluid regimen during PD was associated with significantly higher incidence of POPF (*P* = 0.035) as well as respiratory complications (*P* = 0.027) compared to the restricted and standard fluid regimen. However, the POPF rate was similar in the three groups when BL, grade B fistula, and grade C fistula were analyzed separately because of the small number of patients in each group. Moreover, there was no statistically significant difference in the other outcomes such as postoperative hospital stay, recovery of bowel function, biliary fistula, wound infection, and postpancreatectomy hemorrhage. The overall in-hospital mortality was 7.28%, and the overall postoperative morbidity was 56.95%.

An earlier retrospective analysis by Lindenblatt et al. (2008) [31] revealed no significant association between the incidence of postoperative bleeding (8.2%), wound infection (4.1%), POPF (9.4%), mortality (2.0%), and the volume of IOF administered; the authors concluded that IOF should be targeted at 10–15 ml/kg/hr. However, their analysis included other pancreatic resections along with PD. A study by Boland et al. [32] of 188 patients who underwent PD for adenocarcinoma concluded that the volume of IOC increased with the operative time, blood loss, and intraoperative blood transfusion, but did not correlate with postoperative morbidities. However, a retrospective analysis by Eng et al. [33] showed that high IOF rate was associated with worse perioperative outcomes in patients undergoing PD, especially in those with preoperative serum albumin ≤3.0 g/dl. Another recent study [17] demonstrated that high IOF volume (≥8.2 ml/kg/hr) was associated with an increased incidence of POPF after PD.

The mechanism behind the occurrence of POPF is poorly understood. Recent research [34] suggests that excessive blood loss, pancreatic duct size (<5 mm), soft pancreatic parenchyma, and certain disease pathologies were associated with increased fistula rates. Moreover, two studies [6, 35] showed that excessive intravenous hydration diminishes tissue oxygenation, causes edema, and may cause poor healing of the anastomosis.

The pancreas is a solid organ and lacks distensibility. We believe that a high IOF rate promotes pancreatic and peripancreatic edema formation which hampers healing of pancreato-intestinal anastomosis leading to pancreatic fistula. In contrast, the fistula involving cholangio-intestinal anastomosis and/or gastro-intestinal anastomosis seldom occurs, because both sides of the anastomosis are cavity organs with high distensibility. Besides, the pancreatic anastomosis has a poorer blood supply than cholangio-intestinal anastomosis or gastro-intestinal anastomosis, which makes it more prone to leakage.

Unlike other studies, we demonstrated a statistically significant difference in the incidence of postoperative respiratory complications (*P* = 0.027). Most of these respiratory complications (mainly ARDS and pleural effusion) were seen secondary to grade C POPF and intra-abdominal collections.

However, there are several limitations of this study. First, it is a retrospective study and liable to selection bias. A prospective analysis about the relationship between perioperative fluid management and complications following PD is currently in development which will address this issue. Second, although we found that there was a correlation between IOF volume and some of the outcomes following PD, IOF administration is just a part of perioperative fluid management, and postoperative fluid administration may also play a vital role in the outcomes. Third, several other factors such as pancreatic duct diameter and pancreatic consistency were not studied which could affect the outcomes as seen in previous studies. Lastly, this was a single-center study with a small sample size.

In conclusion, IOF management during PD is crucial and affects the incidence of POPF and respiratory complications. A restrictive fluid therapy may help to prevent those complications. Future randomized clinical trials comparing different fluid regimens are required to validate our hypotheses.

## Figures and Tables

**Table 1:** Clinical characteristics of the study patients (*n* = 151), and comparison of preoperative and intraoperative characteristics among the three intravenous fluid administration rate groups.

Parameter	Total (*n* = 151)	Restricted group (*n* = 47)	Standard group (*n* = 76)	Liberal group (*n* = 28)	*P* value
Age (years)	59.11 ± 10.25	59.11 ± 10.25	56.91 ± 9.76	61.93 ± 10.39	.072
Gender					.209
Male	92 (60.93%)	31 (65.96%)	48 (63.16%)	13 (46.43%)	
Female	59 (39.07)	16 (34.04%)	28 (36.84%)	15 (53.57%)	
Preop ALB	37.94 ± 2.53	38.22 ± 2.55	37.65 ± 2.60	38.23 ± 2.30	.388
BMI	23.95 ± 3.37	23.94 ± 3.41	24.01 ± 3.37	23.80 ± 3.42	.960
Comorbidities	91 (60.26%)	29 (61.70%)	46 (60.53%)	16 (57.14%)	.925
Hypertension	53	17	27	8	
Diabetes	25	9	11	5	
COPD	9	2	6	2	
Others	4	1	2	1	
Preop TBIL	126.02 ± 64.00	135.36 ± 64.84	120.18 ± 51.48	126.19 ± 89.34	.445
IOF volume (ml)	4294 ± 1046	3990 ± 870	4309 ± 1178	4763 ± 736	**.007**
Weight (kg)	62.78 ± 8.24	64.98 ± 7.15	61.87 ± 8.96	61.57 ± 7.42	.087
Duration of operation (hr)	6.12 ± 1.51	6.38 ± 1.54	5.98 ± 1.59	6.02 ± 1.21	.345
IOF rate (ml/kg/hr)	11.89 ± 3.53	8.25 ± 1.22	12.01 ± 1.42	17.68 ± 1.78	**.000**
Tumor type					.687
Malignant	133 (88.08%)	41 (87.23%)	66 (86.84%)	26 (92.86%)	
Benign	18 (11.92%)	6 (12.77%)	10 (13.16%)	2 (7.14%)	

Abbreviations: ALB: albumin; BMI: body mass index; COPD: chronic obstructive pulmonary disease; TBIL: total bilirubin; IOF: intraoperative fluid.

**Table 2:** Details of intraoperative characteristics, postoperative outcome (*n* = 151), and comparison of them among the three groups.

Parameter	Total (*n* = 151)	Restricted group (*n* = 47)	Standard group (*n* = 76)	Liberal group (*n* = 28)	*P* value (restricted vs. liberal)
IOF rate (ml/kg/hr)	11.89 ± 3.53	8.25 ± 1.22	12.01 ± 1.42	17.68 ± 1.78	
Estimated blood loss (ml)	313 ± 238	330 ± 230	285 ± 176	360 ± 364	.304
Patients transfused in PD	16 (10.60%)	3(6.38%)	8(10.53%)	5(17.86%)	
Volume of blood transfusion (ml)	500 ± 146	467 ± 115	500 ± 151	520 ± 179	.897
POF1 rate (ml/kg/hr)	2.08 ± 0.45	2.07 ± 0.44	2.08 ± 0.46	2.08 ± 0.46	.980
POF2 rate (ml/kg/hr)	2.04 ± 0.45	2.03 ± 0.44	2.06 ± 0.47	2.02 ± 0.43	.899
POF3 rate (ml/kg/hr)	2.04 ± 0.44	2.02 ± 0.43	2.08 ± 0.47	1.98 ± 0.41	.575
Postoperative length of stay (days)	19.78 ± 9.79	22.36 ± 11.10	18.82 ± 9.66	18.07 ± 6.78	.088
Recovery of bowel function (days)	4.66 ± 1.35	4.72 ± 1.57	4.62 ± 1.26	4.79 ± 1.20	.829
Drainage of POD1 (ml)	133 ± 112	112 ± 71	140 ± 119	148 ± 144	.286
Drainage of POD2 (ml)	105 ± 162	110 ± 169	102 ± 150	104 ± 187	.962
Drainage of POD3 (ml)	72 ± 150	62 ± 108	76 ± 163	76 ± 178	.867
Complications	86 (56.95%)				
POPF ^[^26^]^	17 (11.26%)	3 (6.38%)	7 (9.21%)	7 (25%)	**.035**
BL	4	1	2	1	
Grade B	6	1	2	3	
Grade C	7	1	3	3	
Biliary fistula	9 (5.96%)	3 (6.38%)	4 (5.26%)	2 (7.14%)	.927
Wound infection	12 (7.95%)	3 (6.38%)	5 (6.58%)	4 (14.29%)	.459
Postpancreatectomy hemorrhage ^[^28^]^	11 (7.28%)	2 (4.26%)	6 (7.89%)	3 (10.71%)	.558
Intra-abdominal infection	10 (6.62%)	2 (4.26%)	5 (6.58%)	3 (10.71%)	.553
Respiratory complication	15 (9.93%)	1 (2.13%)	8 (10.53%)	6 (21.43%)	**.025**
Pulmonary infection	9 (5.96%)	1	3	5	
Pulmonary edema	5 (3.31%)	0	1	4	
Pulmonary embolus	1 (0.66%)	0	1	0	
Delayed gastric emptying ^[^29^]^	5 (3.31%)	1 (2.13%)	2 (2.63%)	2 (7.14%)	.456
Chylous fistula ^[^27^]^	3 (2.65%)	0	2 (2.63%)	1 (0.77%)	.485
Liver abscess	2 (1.32%)	1 (2.13%)	1 (1.32%)	0 (0)	.738
Others	2(1.32%)	0	1 (1.32%)	1 (0.77%)	.738
Mortality (in-hospital)	11 (7.28%)	2 (4.26%)	4 (5.26%)	5 (17.86%)	.057
POPF/intra-abdominal infection	6	0	2	4	
Hemorrhage	3	1	1	1	
Pulmonary embolus	1	0	1	0	
Acute renal failure	1	1	0	0	

Abbreviations: IOF: intraoperative fluid; POF: postoperative fluid; POD: postoperative day; POPF: postoperative pancreatic fistula; BL: biochemical leak.

**Table 3:** Multivariate regression analysis: factors affecting complications.

		Pancreatic fistula			Respiratory complication		
	Odds ratio	95% CI	*P* value	Odds ratio	95% CI	*P* value	
IOF rate (ml/kg/hr)	5.195	1.142–23.823	**.023**	7.302	0.676–58.231	**.025**
Preop ALB	19.053	3.048-11.126	**.000**	15.967	1.378-118.636	**.000**
BMI	18.658	2.892-10.301	**.000**	17.055	1.626-133.721	**.000**
Estimated blood loss (ml)	0.555	.063–4.874	.596	0.271	.033–2.613	.602
Duration of operation (hr)	1.278	.234–6.983	.777	0.711	.092–7.057	.399
POF1 rate (ml/kg/hr)	0.805	.50–13.067	.878	0.906	.101–9.431	.341
POF2 rate (ml/kg/hr)	4.812	.410–56.425	.211	1.925	.292–27.465	.165
POF3 rate (ml/kg/hr)	0.486	.052–4.569	.528	0.166	.023–2.336	.684
Benign/malignant	0.269	.069–1.055	.060	1.555	.163–10.712	.212
Pancreatic fistula				91.384	10.359-901.33	**.000**

Abbreviations: IOF: intraoperative fluid; POF: postoperative fluid; ALB: albumin; BMI: body mass index.

## Data Availability

The datasets generated and analyzed during the present study are available from the corresponding author on reasonable request.
